# Reconstruction of Protein Backbones from the BriX Collection of Canonical Protein Fragments

**DOI:** 10.1371/journal.pcbi.1000083

**Published:** 2008-05-23

**Authors:** Lies Baeten, Joke Reumers, Vicente Tur, François Stricher, Tom Lenaerts, Luis Serrano, Frederic Rousseau, Joost Schymkowitz

**Affiliations:** 1 SWITCH Laboratory, Vrije Universiteit Brussels, Brussels, Belgium; 2 VIB, Vrije Universiteit Brussels, Belgium; 3 Center for Genomic Regulation, Barcelona, Spain

## Abstract

As modeling of changes in backbone conformation still lacks a computationally efficient solution, we developed a discretisation of the conformational states accessible to the protein backbone similar to the successful rotamer approach in side chains. The BriX fragment database, consisting of fragments from 4 to 14 residues long, was realized through identification of recurrent backbone fragments from a non-redundant set of high-resolution protein structures. BriX contains an alphabet of more than 1,000 frequently observed conformations per peptide length for 6 different variation levels. Analysis of the performance of BriX revealed an average structural coverage of protein structures of more than 99% within a root mean square distance (RMSD) of 1 Angstrom. Globally, we are able to reconstruct protein structures with an average accuracy of 0.48 Angstrom RMSD. As expected, regular structures are well covered, but, interestingly, many loop regions that appear irregular at first glance are also found to form a recurrent structural motif, albeit with lower frequency of occurrence than regular secondary structures. Larger loop regions could be completely reconstructed from smaller recurrent elements, between 4 and 8 residues long. Finally, we observed that a significant amount of short sequences tend to display strong structural ambiguity between alpha helix and extended conformations. When the sequence length increases, this so-called sequence plasticity is no longer observed, illustrating the context dependency of polypeptide structures.

## Author Summary

Large-scale DNA sequencing efforts produce large amounts of protein sequence data. However, in order to understand the function of a protein, its tertiary three-dimensional structure is required. Despite worldwide efforts in structural biology, experimental protein structures are determined at a significantly slower pace. As a result, computational methods for protein structure prediction receive significant attention. A large part of the structure prediction problem lies in the enormous size of the problem: proteins seem to occur in an infinite variety of shapes. Here, we propose that this huge complexity may be overcome by identifying recurrent protein fragments, which are frequently reused as building blocks to construct proteins that were hitherto thought to be unrelated. The BriX database is the outcome of identifying about 2,000 canonical shapes among 1,261 protein structures. We show any given protein can be reconstructed from this library of building blocks at a very high resolution, suggesting that the modelling of protein backbones may be greatly aided by our database.

## Introduction

High-resolution structure determination of proteins and protein complexes via experimental methods occurs at a significantly slower pace than the collection of novel protein sequences. As a result, less than 30% of human proteins have a known structure in the Protein Data Bank and the percentage for other species is significantly lower [1]. In addition, structures mostly cover one or a small number of protein domains, thus covering only a fraction of the total sequence of the protein. Homology modeling improves this coverage using related proteins with known structures to build a model [2]–[5]. The construction of an adequate homolog can be divided into two related tasks: the placement of the amino acid side chains on a given backbone template and the detection of changes in backbone conformations that are required to accommodate the new sequence. For proteins that are relatively close in terms of sequence identity, the backbone-modeling problem is usually ignored, but in many cases the best homology template shows less than 50% homology with the target, and small compensatory changes to the backbone are likely to be required to obtain an accurate model. Recent advances in protein backbone modeling are based on the observation that protein structures are built from a finite repertoire of structural folds [6]. Structural redundancy allowed the classification of protein folds such as in the SCOP database [7], the CATH database [8] or the FSSP classification [9],[10]. The unit of fold classification is usually a protein domain, since large proteins are generally composed of multiple domains. As a consequence, the classification comprises a hierarchical organisation of protein domains that embodies evolutionary and structural relationships. By creating more categories and thus refining the secondary structure descriptions, it has been proposed that a set of discrete backbone conformational states can be derived [11],[12]. Different research groups demonstrated the usefulness of such fragment libraries when reconstructing protein structures by generating sets of protein decoys [13]–[17]. In the latest editions of CASP, prediction approaches that assemble fragments of known structures into a candidate structure have proven to be successful [17]–[20]. In fragment assembly methods, the assumption is made that local interactions create a particular conformational bias, but do not uniquely define local structure [21]–[24]. Instead, environmental constraints will determine the overall compact protein conformation. The construction of a final model is composed of three steps: The first step involves a selection of fragment candidates based on their stability that can be measured by a simplified scoring function [25]. In the second step the fragments are assembled combinatorially [26],[27]. In the final step the obtained structure is optimized through the employment of a force field [27],[28]. This method works well for small *all* *α* class proteins, and reasonably well for *α*/*β*, *α*+*β* and *all* *β* class proteins. The fragment approach has been successfully applied in the structure prediction algorithm Rosetta of Baker and co-workers [29]–[31], which also proved to be successful in accurately designing new folds [32]. Publicly accessible libraries however are limited; they are typically small and consider lengths between 4 and 7 residues. For instance, by examining fragments of 5 residues, Kolodny and Levitt [6] created a library of 20 fragments, while Etchebest found only 16 building blocks of this length [33]. The alphabet of Camproux [34] consists of 27 structural classes and is based on motives of 4 residues. By employing their hypercosine method on a set of 150,000 length-7 protein fragments, Hunter and Subramaniam [35] discovered 13 minimal centroids or representative fragment shapes found in proteins at a resolution of 0.80 Angstrom. As such low resolution approaches, restricted to a single fragment length and thus resulting in a limited set of building blocks, might constitute an advantage in terms of computational efficiency for *ab initio* structure prediction methods, it will also lead to a significant loss of information. Wainreb et al made it possible to cluster variable sized fragments, consisting of at least 15 residues, through the implementation of their SSGS algorithm [36]. By allowing more variability in the alignment of loop locations, they created a library of 8,933 building blocks. An alternative approach, as implemented by DePristo et al [37], uses an ensemble of artificially generated small polypeptide conformations instead of sampling conformations from known protein structures. By constraining the chemical properties such as the idealized geometry, phi/psi angles and excluded volume they constructed ensembles of near-native conformations consistent with a surrounding fixed protein structure. Our strategy focuses on obtaining a comprehensive set of high-resolution structural fragments without using artificial data or restricting fragment lengths. We decided to partition a non-redundant set of high-resolution protein structures into fragments that consist of 4 to 14 residues, because preliminary tests indicated the lack of high structural similarity for more than 50% of all fragments when larger lengths were considered. Subsequently, clustering techniques were employed to identify structural motifs that are recurrent in different protein structures. Over 1,000 recurrent fragment structures or classes were found for each considered peptide length when a structural variation proportional to the length of the fragment (0.1 Angstrom per residue) was allowed. As suggested in [6],[38], it is important to determine how well the classes of the fragment library cover fold space in order to estimate its value. When applied to protein structures not used in the construction of the database, this coverage turned out to be 99% on average using a 1 Angstrom RMSD threshold. The latter implies that in the majority of the cases studied the so-called irregular regions or loops can also be reconstructed from recurrent building blocks. Through the employment of a global fit reconstruction algorithm, backbone traces were generated having an average accuracy of 0.48 Angstrom RMSD.

Additionally, the ability to use BriX for local secondary structure prediction was examined by looking at the sequence-structure relationship within classes. According to previous findings [25], the sequence conservation within classes was rather low because of the large number of determined building blocks originating from different families. Nonetheless, this analysis led to a quantitative illustration of the context-dependence of polypeptide structure. A significant amount of small sequences tend to display strong structural ambiguity: for fragments of length 5, 14% of the fragment pairs with identical sequences have structural difference within the range of a helix-to-sheet jump. These so-called plastic sequences, i.e. sequences that display diverse structural conformations, display a strong preference for the aliphatic residues Alanine, Valine, and Leucine. For fragments of more than 5 residues sequence plasticity is no longer observed, showing that the need for additional context to determine secondary structure is much reduced for longer fragments.

## Results/Discussion

**Figure 1:**
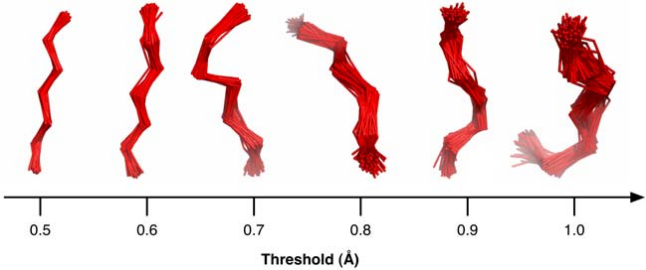
Effect of varying RMSD on structural variation within a class. The plot shows the fragment content of equivalent BriX classes of length 7 created with fixed RMSD thresholds from 0.6 to 1 Angstrom. The increase in structural variation with higher RMSD thresholds is not uniformly distributed over all positions; there is a clear tendency towards the terminal positions (both carboxy- and amino-terminal), resulting in a fan-like arrangement.

**Figure 2:**
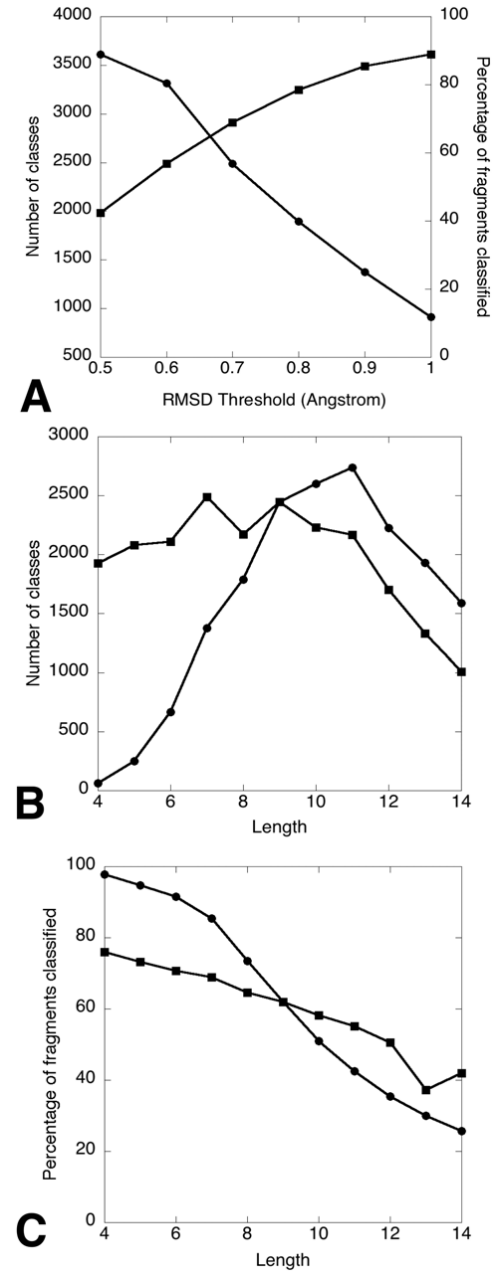
BriX clustering statistics. (A) Effect of increasing RMSD threshold. Shown is the number of BriX classes (circles) and the percentage of classified fragments (squares) in function of the RMSD threshold (0.5–1.0 Angstrom) used during the clustering for fragments containing 7 residues. As expected, higher thresholds result in fewer fragment classes and more identified recurrent fragment structures as the variation within a class is higher and a class thus contains more elements. A threshold of 0.6 Angstrom is sufficient to classify more than half of all fragments of length 7. (B) Number of classes for varying fragment lengths. Shown is the number of classes in function of the fragment length clustered with a fixed RMSD threshold (circles) of 0.9 Angstrom and a RMSD proportional to the fragment length (squares), by increasing the RMSD with 0.1 Angstrom per residue. In both figures, the number of classes increases with the length until a turning point is reached, after which the number of classes drops steeply. When a fixed RMSD is applied, this turning point clearly occurs at fragment length 11, reaching the level of 2,740 classes. (C) Percentage of classified fragments for varying fragment lengths. Shown is the percentage of classified fragments in function of the fragment length clustered with a fixed RMSD threshold (circles) of 0.9 Angstrom and a RMSD proportional to the fragment length (squares), by increasing the RMSD with 0.1 Angstrom per residue. In both plots, the number of classified fragments smoothly decreases when larger fragment lengths are considered. When a proportional RMSD is applied, this decrease is less steep, resulting in a classification percentage of more than 40% compared with 26% (fixed RMSD) at fragment length 14.

By sliding a window of varying length (4–14 amino acids) over a non-redundant set of 1,261 high quality protein structures retrieved from the WHAT IF software package [39], about 260,000 protein fragments of each length were obtained. Using a multi-step clustering approach (see Materials and Methods section), these fragments were clustered into more than 1,000 up to approximately 2,000 structural classes, for each length ranging from 4 to 14 residues. Furthermore, we distinguished different degrees of variation inside the classes, by performing the clustering with 6 different distance thresholds. For instance, the considered RMSD thresholds for fragments consisting of 7 residues were 0.5, 0.6, 0.7, 0.8, 0.9 and 1.0 Angstrom. Clustering with varying RMSD thresholds was performed to provide degrees of structural diversity that are suited for a wide range of modeling requirements. Particularly for homology modeling, the threshold variation is a useful parameter for modeling structures with varying sequence identity. Lower thresholds yield more accurate fits for high identity regions, while larger thresholds enable the modeling of loops or other regions with lower sequence identity. Larger thresholds often result in fanlike shapes at the end of the fragments and at local loop positions. Figure 1 illustrates the structural variation within equivalent classes comprising 7 residues constructed at different. Increasing the distance threshold used to cluster the fragments resulted in a decrease in the number of classes being identified as regular structures. The number of classified fragments, i.e. fragments belonging to a fragment class, increased with larger thresholds applied in the clustering process (see Figure 2A). A larger threshold implies a wider radius around the class centroids, and thus larger and fewer classes with more internal variation. Monitoring the number of classes in function of the length of fragments classified at a fixed threshold (RMSD of 0.9 Angstrom, Figure 2B), we observed an increase in the number of classes with length until a fragment length of 11 residues, after which the number of classes dropped steeply (length 12–14). This turning point at fragment length 11 was not observed when plotting the percentage of fragments classified at RMSD threshold 0.9 versus the fragment length (Figure 2C). Here a smooth decline of the number of classified fragments for increasing fragment lengths was observed. When the same analysis was performed on clustering results for RMSD thresholds proportional to fragment length (0.1 Angstrom variation per residue), thus allowing more variation for larger fragment lengths, a similar pattern applied. Although less steep, we still observed a decrease of the percentage of classified fragments with increasing fragment length (Figure 2C). Again, a turning point (at fragment length 9) after which the number of classes dropped was observed (Figure 2B), albeit at a different fragment length then when a fixed threshold was applied. These results indicate that increasing the clustering threshold for longer fragments did not suffice to construct the same number of fragment classes as at shorter lengths. Increasing fragment length results in a larger conformational space and variability of classes, impairing the clustering of fragments into well-defined classes.

### The Building Blocks

**Figure 3:**
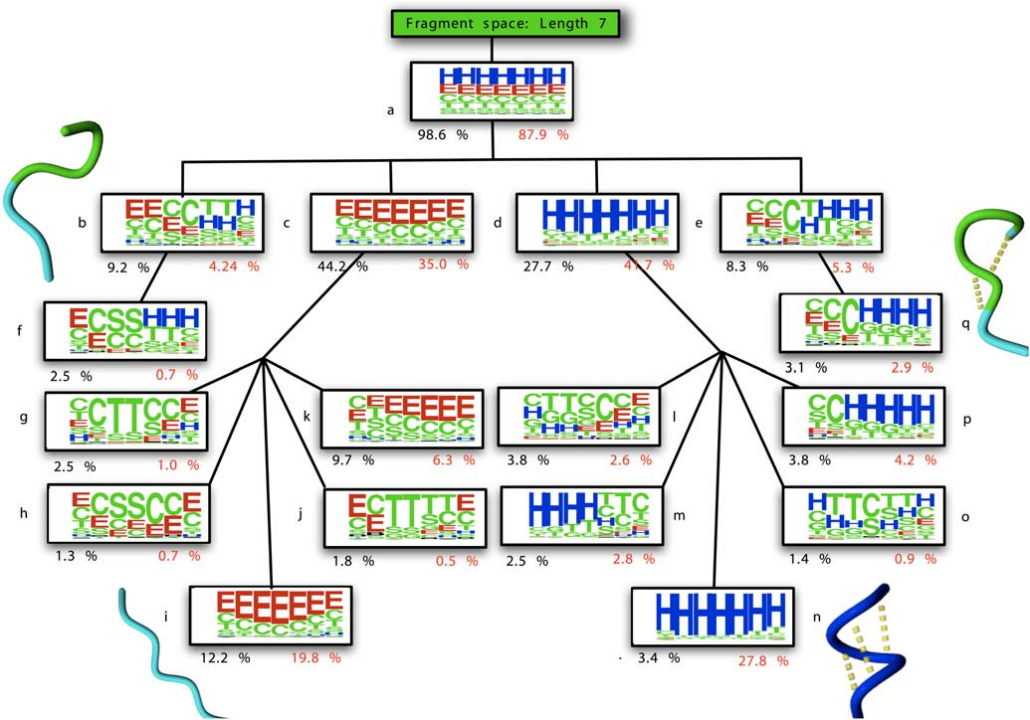
Structural hierarchy of classes based on RMSD distance. The nodes are represented by means of a DSSP logo (generated using WebLogo [47]) and a denotation of the percentage of BriX classes (in black) and fragments (in red) it contains. At the second level, the hierarchical clustering is able to distinguish the two major secondary structure elements: strands and helices. These branches are further partitioned into loops and small turns. Notable is the content difference between the *pure* secondary structure nodes (*k* and *p*) at the bottom level of the tree. Although node *k* consists of 12.2% of all BriX classes, it only represents 19.8% of the fragments of the WHAT IF set. Node *p*, on the contrary, embodies 27.8% of the fragment space, while holding only 3.4% of the BriX classes. This discrepancy shows that the stronger structural constraints imposed on helices result in fewer and larger helical classes than the strand classes created with the same threshold.

**Figure 4:**
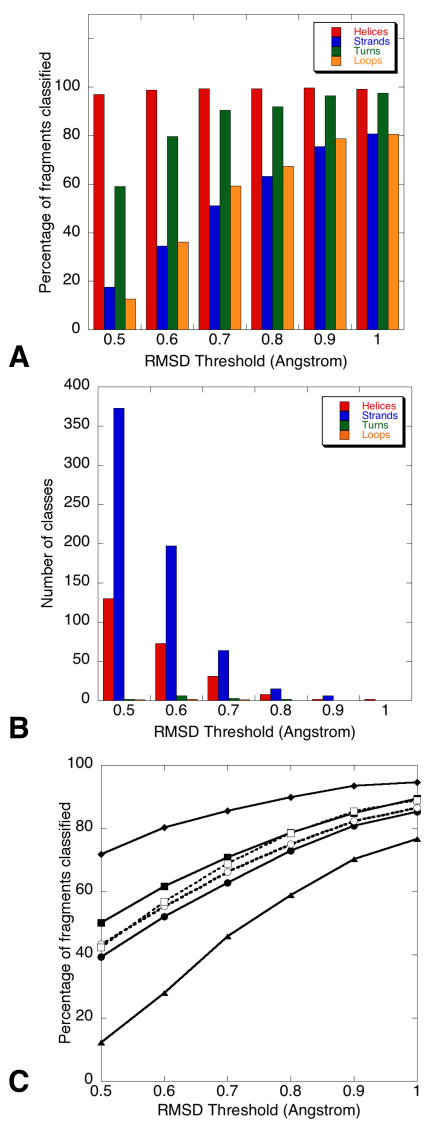
BriX statistics with regard to secondary structure content. (A, B) Effect secondary structure on the respective classification. The plots show data for classes consisting of one secondary structure element, i.e., pure helical (red), strand (blue), turn (green), and loop (orange) classes. The data selection was based on the fragments or fragment classes having an overall DSSP content of more than 80% in these 4 structural elements. Shown is the percentage of classified fragments regarding an increasing distance threshold. Although the vast majority of *helical* fragments were found to be recurrent (A), the number of respective structural classes is low compared to the number of *strand* classes (B). Because of the stabilizing hydrogen bonds, helices do not allow a lot of variation, resulting in few large BriX classes. The variable character and infrequent occurences of loops and turns are the main reason for the small number of recurrent structures and poor classification results. (C) Classification results for the Astral40 validation test. The BriX fragment classification obtained from the WHAT IF globular structure set was used to classify fragments generated from the Astral40 structures. Experiments evaluating the effect of increasing threshold on the percentage of classified fragments were repeated for the full Astral40 set (open circles) and for the Astral40 structures of the major SCOP classes (all α [diamonds], all β [triangles], α/β [closed circles], and α+β [squares]). The initial classification results for the WHAT IF generated fragments (open squares) are shown for reference. The full Astral set follows a similar classification pattern as the WHAT IF set, showing that the latter gives a good representation of protein structures in general. The higher classification rate of helical proteins points to a lower structural variation within these structures.

The dendogram in Figure 3 is the result of applying a clustering approach similar to the one used to construct the BriX database on the class centroids comprising 7 residues. In this way, the fragment space is rebuilt by grouping the BriX classes into *superclasses* based on root mean square distances between the class centroids. For reasons of simplicity only the largest classes (i.e. superclasses that contain more than 1% of BriX classes) are shown on each level. At the top of the classification, one branch is shown that comprises 98.6% of all classes and 87.9% of all fragments allowing a maximal distance of 1.8 Angstrom RMSD between the class centroids. At the second level the clustering method is capable of separating the two principal secondary structure elements: strands and helices. These segregrate further into smaller, more specific conformations. A counterintuitive result is that the clustering method does not differentiate between *turn* and *helix* secondary structure elements on the top level. Instead we find them at different levels in the tree (see Superclasses *i*, *m*, *n* and *q*). Figure 4A shows the percentage of fragments that were found to be recurrent regarding an increasing distance threshold. Clearly shown is the difficulty to classify *sheets* and *loops*, despite an increasing distance threshold. This is because they do not exist in well-defined conformations, but instead occupy a wide range of geometries. As a consequence, only a few recurring structures could be identified, resulting in a low number of classes and a large number of unclassified *sheet*- and *loop* fragments. This was also observed by Du et al [40] when determining the probability of finding a short protein fragment back among non-homologous structures in the Protein Data Bank. According to Figure 4A, the authors perceived a nearest neighbor RMSD distribution for β fragments close to that for loop fragments.

Further analysis revealed the tendency of *turns* to become associated with *helix* classes when larger distance thresholds were applied. Another observation, as can be seen in both Figure 3 and Figure 4B, was the small amount of structural variants found in the helical classes, resulting in a low number of identified *helix* classes, while the vast majority of the *helical* fragments were classified. *Strand* fragments, on the other hand, exhibited a lot more structural variation, resulting in a significantly larger number of smaller classes.

Figures S1A and S1B show the frequency of the four main secondary structure elements inside the BriX classes comprising 10 and 7 residues. The DSSP (Dictionary of Protein Secondary Structure [41]) secondary structure assignments for the four main secondary structure elements (helix, sheet, turn and loop) were counted and plotted against the percentage of classes with a similar composition. Clearly shown is the occurrence of *turns* and *loops* in 1 to 4 residue patterns, whereas *helices* and *strands* take up longer stretches within the fragment (peaks at 5 and 6 residues respectively). Classes of 10-residue long fragments revealed a substantial heterogeneity, as the presence of pure classes is below 5%. For fragments consisting of 7 residues, on the contrary, we observed a percentage of nearly 25% pure helix classes and 5% pure sheet classes.

### Validation Tests on the BriX Fragment Classes

The relevance of the created fragment classes in the BriX database was evaluated by two validation tests based on 7,290 high resolution protein structures taken from the Astral set with less than 40% internal structural homology [42]. In the first test, the generated Astral fragments were classified into the existing BriX class hierarchy to assess whether the vocabulary of fragments obtained from the WHAT IF structure set was sufficient to describe the Astral40 structure set. In the second test the fragment classes were used to reconstruct the backbones of all known human structures in the Protein Data Bank (PDB) using a novel backbone coverage algorithm.

In the first test, a special focus was directed towards the classification differences of *α*-, *β*-, *α*/*β*- and *α*+*β* proteins. In addition to removing sequence redundancy to exclude homologs, the test set was constructed following the structural hierarchy of SCOP [7]. Figure 4C illustrates the potential of BriX to give a good representation of protein structures in general. Although the full Astral set followed a classification pattern similar to the WHAT IF set, a clear correlation with the fold was noted. In accordance with the previous finding of [35], SCOP classes with higher helical content reached a higher level of classification (from 70% up to 95%) than those mainly composed of sheets (from 10% up to 70%). As there was less variation within helical classes, more fragments were classified at lower thresholds. At higher thresholds the difference between the folds diminished.

**Figure 5:**
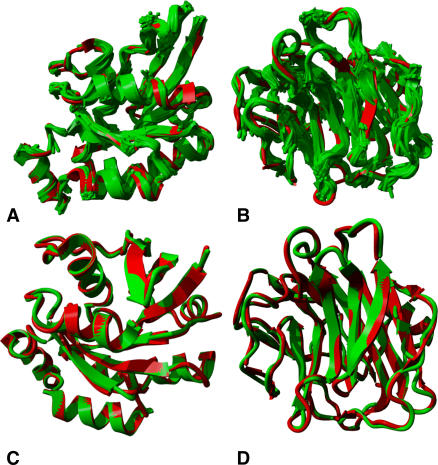
Reconstruction of human protein backbones using BriX classes. (A, B) Local fit approximation for the reconstruction of the set of human protein structures: some examples. The backbones (in red) of α G25K GTP-binding protein (A) and β human C-reactive protein (B) fully covered with BriX classes (green). The covering algorithm selected 35 and 40 redundancy filtered fragment classes to describe the respective structures. (C, D) Global fit approximation for the reconstruction of the set of human protein structures: some examples. A backbone trace of α G25K GTP-binding protein (C) and β human C-reactive protein (D). The target proteins are shown in red and the approximations are shown in green. The overall RMSD is 0.4542 Angstrom and 0.5614 Angstrom, respectively.

In order to assess the accuracy of a fragment library to describe known protein structures two different measures have been proposed in previous works [6],[34]: the local fit and global fit approximation. The first measure determines how well each fragment of a protein structure can be locally approximated by the best corresponding fragment class. Note that in this test it is not required to assemble the fragments to obtain a unique backbone trace. In addition, we calculated the total percentage of the structure that could be covered by the fragment classes (see Materials and Methods section). For reasons of generality, the validation test considered a representative set of human proteins, extracted from the PDB database (see Materials and Methods section). This relatively small set contained 935 structures, equivalently balanced over the existing folds (as is illustrated in Table 1). In order to fully consider the secondary structure differences, separate tests were carried out for α (*A*) proteins, β (*B*) proteins, α and β (*A*/*B* and *A*+*B*) proteins, according to the SCOP classification. With an average RMSD of 0.16 Angstrom for the local fit approximation BriX improves the previously obtained 0.23 Angstrom RMSD by Camproux et al using 27 structural classes [34]. Kolodny and Levitt achieved an average RMSD of 0.26 and 0.39 Angstrom for respectively 4 and 14 classes considering fragments of four-residue length [16], The 16-states alphabet describing fragments of 5 residues of De Brevern et al [43] approximated the local structure with an accuracy of 0.51 Angstrom. Furthermore, Table 1 shows BriX achieves a coverage of 99 to 100%. Figure 5 illustrates an all α (5A) and an all β (5B) class protein, originating from the human proteins validation set, entirely covered by BriX classes. Remarkable is that even all β proteins and irregular structures such as *loops* appeared to have full coverage of BriX classes. This implies that in spite of their hypervariable character, loops are made up of regular building blocks.

**Table 1:** Coverage of human proteins with BriX classes.

SCOP Classification	Number of Structures	Average Coverage, %
α proteins	216	99.88
β proteins	361	99.96
α and β proteins (α/β)	78	99.90
α and β proteins (α+β)	124	100
Remaining proteins	156	99.97

Results of the second validation test on the human proteins set, showing the average fraction of a protein structure that can be covered with BriX classes for the major SCOP classes. The high coverage rates achieved prove the ability of BriX to describe an arbitrary protein structure.

The second measure to qualify a fragment library is the global fit approximation in which a whole protein is reconstructed using the BriX fragment classes. By employing a depth-first search algorithm (see Materials and Methods section) backbone traces for the human proteins were generated (see Figure 5C and 5D). To avoid ambiguity we repeated this experiment on the Park & Levitt set [44], which has also been considered during previous reconstruction attempts [6],[34]. Although the calculation of the best global fit approximation was computationally too expensive, we achieved an average accuracy of 0.48 Angstrom RMSD for the resulting backbone reconstructions (see Figure S2A). In order to obtain this accuracy, the fragment classes from all lengths were considered. On the same protein set, Camproux et al [34] managed to obtain an average accuracy of 0.64 Angstrom RMSD, while Kolodny et al [6] achieved 0.92 Angstrom RMSD. Interesting observations were made during the different validation tests. As larger fragment lengths can describe regular secondary structure elements more accurately, loop/turn regions were best approximated by shorter fragments, containing 8 residues and less. This result was most pronounced for the all-α SCOP class (see Figure S2B and S2C). This illustrates the benefit of using a fragment library not restricted to one fragment length. One of the bottlenecks in predicting protein structures is the relative spatial organization of regular secondary structure elements.

To address the applicability of our fragment library, we looked more in detail into the bridging region between those regular secondary structure elements and the loop/turn region. In previous studies [40],[45] loop structures were considered as seven-residue fragments with less than four continuous α-helical or β-strand residues as defined by DSSP. In this experiment loops are defined as in [40],[45]. As loops occur in a wide range of lengths, we slightly adapted the loop definition into a part of a protein structure consisting of at least 4 residues without 4 continuous α-helical or β-strand residues. The experiment consisted in finding local matches for the regions between a regular secondary structure element and a loop region. A special focus was directed towards the fragment length of these matches when forcing the search process to include two residues from the regular side. The search algorithm was able to find at least one match for each region by considering a threshold of 1.0 Angstrom RMSD, According to our previous results, Figure S3A and S3B show that these regions are best approximated by smaller building blocks.

The various results obtained in this second validation step suggest that although not every short sequence in a protein encodes a regular structure, the total protein structure is built with small building blocks. The size of the building block needs to describe a small region is dependent on the type of secondary structure elements present in that region. Hypervariable regions such as loops are composed of multiple smaller regular building blocks, while a single longer BriX class can often describe entire helices and strands.

### Plastic Sequences

**Figure 6:**
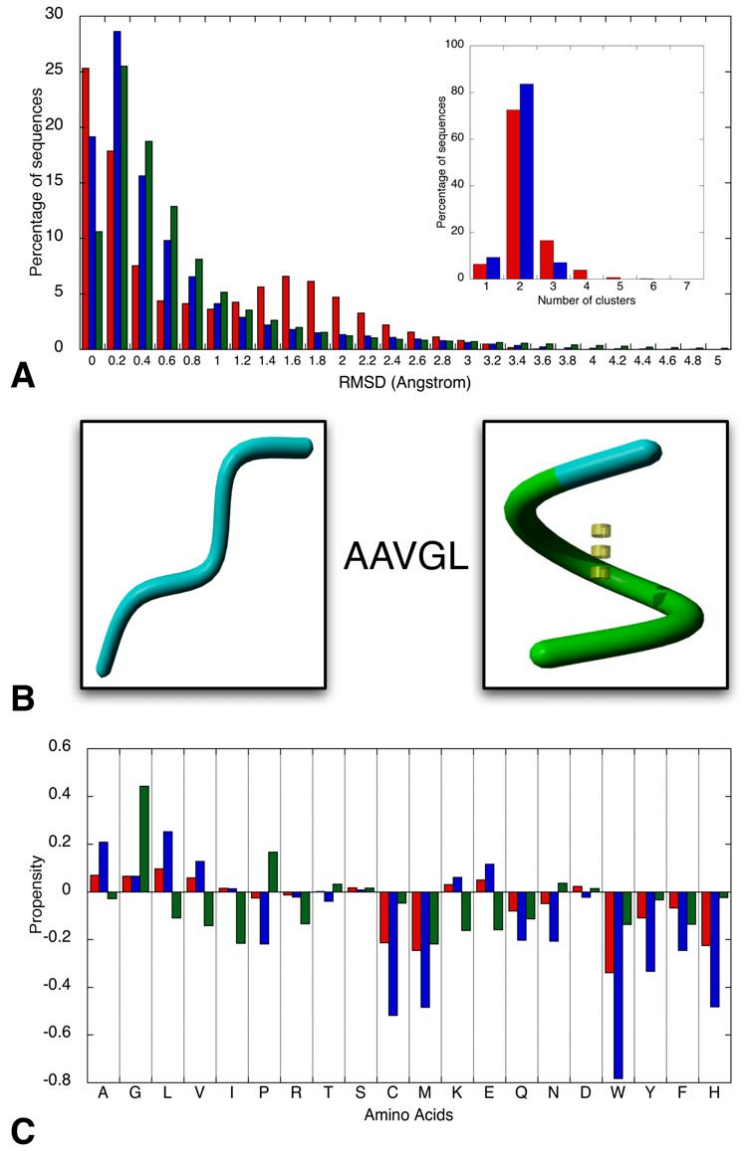
Presence of structural switches within groups of fragments containing identical residue sequences. (A) The effect of the fragment length on the structural variation. Shown is the percentage of identical sequence pairs in function of the structural distance between them for fragments of length 5 (red), 9 (blue), and 13 (green) in the Astral40 dataset. Clearly shown in the main histogram is the tendency of smaller fragments to manifest large structural variation. The smaller plot is the result of carrying out the Hierarchical Agglomeration process on nearly 40,000 sequences where this variation was recognized. The clustering considered two different distance thresholds: 1.5 Angstrom (red) and 2.0 Angstrom (blue) RMSD. The plot shows that for the vast amount of these sequences, 2 structural groups can be identified. (B) Example of structure differences for one amino acid sequence. The sequence *AAVGL* can adopt both a strand (left) and helix (right) conformation. The strand conformation is present in the Antigen 85-C protein (structure 1DQZ) and starts at residue-number 119. The helical conformation is cut from the 2,2-dialkylglycine decarboxylase protein (1D7U) at residue-number 311. (C) Amino acid usage in plastic sequences. Shown is the frequency of amino acids occurring in sequences that only allow small structural jumps, resulting in tiny variations of a certain conformation (in red) and in sequences where these jumps are larger, resulting in drastic structure switches (in blue). The green bars indicate the presence of the respective amino acids in fragments that were left unclassified in BriX classes, due to their irregular character. Three groups can be distinguished: amino acids promoting (1) a single-well defined regular structure (such as Tryptophan, Tyrosine, Phenylalanine, Cysteine, Asparagine, and Methionine), (2) several regular structures or structural jumps (such as Alanine, Leucine, and Valine), and (3) irregular structures (such as Glycine and Proline).

When considering part of a protein structure, the possibility was examined to predict the corresponding BriX class from sequence information. However, exceptions aside, the overall sequence conservation within the classes was rather low, precluding sequence to structure prediction. This is to be expected due to the large number of classes resulting from the high-resolution clustering. In addition, an analysis was carried out to identify the magnitude of structural variance in conformations that a single sequence can adopt. The experiment, from which the results are shown in Figure 6, consisted of calculating the pairwise RMSD between fragments with an identical sequence (see Materials and Methods section). Figure 6A shows the normalized distribution of the obtained RMSD values, for three different fragment lengths. Two peaks were observed: the first peak at 0.2 Angstrom revealed that the majority of the fragments, containing an identical amino acid sequence, adopt a similar conformation. A smaller yet significant second peak was recognized at an RMSD of 1.6–2 Angstrom. The idea arose that, certainly for smaller fragment lengths (smaller than 7 residues), a drastic structural switch can occur. This idea was verified through a thorough cluster analysis on nearly 40,000 sequences where this second peak was recognized. For each sequence, the analysis consisted in carrying out the hierarchical agglomeration process on the fragments sharing this sequence. The resulting histogram, shown in Figure 6A, confirms that for the vast amount of these sequences two structural groups could be observed. As the sequence length increases, the second peak in Figure 6A gradually disappears, indicating that additional structural context information is required to remove structural ambiguities.

A closer look at the residues that take part in these structural switches revealed the propensity of smaller hydrophobic residues, such as Alanine, Leucine and Valine, to be involved in ambiguous sequences. Remarkably, as Figure 6C clearly indicates, for some residues (Proline, Cysteine, Methionine, Glutamine, Asparagine, Tryptophan, Tyrosine, Phenylalanine and Histidine) this situation seldom arose. Interestingly Proline and Glycine tended to occur in fragments that are not represented in BriX, due to their irregular nature. These so-called structure breakers are inclined to disrupt secondary structures and often give rise to loop or turn-regions. Consequently, three groups could be distinguished with amino acids promoting: 1) a single-well defined regular structure (such as Tryptophan, Tyrosine, Phenylalanine, Cysteine, Asparagine and Methionine), 2) several regular structures or structural ambiguity (such as Alanine, Leucine and Valine) and 3) irregular structures (such as Glycine and Proline).

Another observation was the magnitude of the structural ambiguity: a switch from a perfect α helix to a β strand was recurrently seen. In the case of 5-residue long sequences, for instance, 15% of all observed structural *jumps* invoked such a dramatic switch. In Figure 6B an example is shown of a sequence (AAVGL) that adopted both a perfect sheet conformation (present in the α-chain of the *1DQZ* pdb-file) and a helical conformation (present in the α-chain of the *1D7U* pdb-file) depending on the context in which it was placed.

### Database Access

In order to use the fragment database, the BriX classes are made accessible at http://brix.vub.ac.be through both a search and a browse interface. Depending on the query, the information is displayed in either a *Class view* or a *Fragment view* and arranged in information tabs. As well as information about its content, the Class view offers a sequence alignment view through the web applet *JalView* [46]. With the help of *WebLogo* [47] both a sequence and a DSSP logo are generated. The Fragment view includes a detailed description of the source of the particular fragment, the sequence and the DSSP assignment. In addition, an overview is presented with links to the BriX classes to which the fragment belongs for different distance thresholds. Furthermore, the web applet *JMol* [48] presents an interactive view of the protein fragment.

The search interface allows searching for fragments or classes using specific identifiers (length, sequence information, pdb filename, …). In order to perform a more complex search, an option is present to use regular expressions when describing an identifier. When looking for clearly defined secondary structure classes, the percentage of helices, turns, strands or irregular structures occurring in a BriX class can be specified. The outcome can be sorted according to a user-defined property.

Through the browse interface, it is possible to browse the secondary structure elements present in the BriX classes. A hierarchy is built using the DSSP profiles of the classes. After choosing a branch, intermediate results can be retrieved, resulting in a list of relevant classes.

### Conclusion

In this study we have derived a fragment alphabet, containing fragments from 4 to 14 residues long. Using a multi-step clustering approach more than 1,000 recurring protein structures were identified among 260,000 protein fragments for each length. Through the employment of a global fit method we showed that BriX approximates native structures with an RMSD of 0.48 Angstrom, for all major SCOP classes. Loop regions that appear irregular at first glance could be entirely reconstructed from smaller building blocks, between 4 and 8 residues long. Regular secondary structures, on the other hand, were best approximated by larger building blocks, as they provided closer fits. Regarding the obtained coverage results, we believe that this collection of protein fragments can be employed for various modeling purposes, including loop-modeling applications.

In addition, a sequence analysis revealed the presence of strong structural ambiguity for a significant amount of small sequences. Examining the residues that take part in these so-called plastic sequences, three groups could be distinguished: Amino acids promoting (1) a single-well defined regular structure, (2) several regular structures and (3) irregular structures. When fragment length was increased, sequence plasticity was no longer observed, illustrating the context-dependency of polypeptide structures.

## Materials and Methods

### Construction of the BriX Database

The database of fragments was built using a list of proteins available in the WHAT IF database of high-quality structures [39]. The compact set of high-resolution proteins embodies the specificity present in the SCOP classes, as shown in Table 2, thereby avoiding a bias towards a specific fold.

**Table 2:** Representation of SCOP classes in the WHAT IF structure set.

SCOP Classification	SCOP, %	WHAT IF, %
All α proteins	13	14
All β proteins	21	20
α and β proteins (α/β)	26	33
α and β proteins (α+β)	24	24
Multi-domain proteins (α and β)	3	2
Membrane and cell surface proteins and peptides	2	1
Small proteins	4	4
Coiled coil proteins	2	1
Low-resolution protein structures	4	0
Peptides	1	0
Designed proteins	0	0

By sliding a window of 4 to 14 residues long over the main chain of the proteins all consecutive overlapping fragments were generated. For each length, a clustering process was employed to identify equivalent structures. To avoid performance problems, this process incorporated a pre-clustering stage by grouping the fragments by their secondary structure assignment according to the DSSP [41]. A fragment was described as a sequence of the three-dimensional coordinates of the backbone atoms (*N*, *C*_α_, *C*_β_, *O*) of each residue. Applying the fast RMSD calculation method of Wolfgang Kabsch [49],[50], distance matrices were constructed for each DSSP group. Subsequently, recurrent structures were detected through the employment of the *Hierarchical Agglomeration* algorithm [51] on the distance matrices. Inspection of the structural classes thus obtained revealed the need for a second phase of clustering in which similar subgroups needed to be identified and merged together. To this end, the representative fragment of each subgroup was determined (termed *centroid*). As a consequence, a new distance matrix was generated composed of the pairwise RMSD distances between the centroids. Once more, Hierarchical Agglomeration was applied to the matrix to detect close subgroups that could be joined, resulting in a final collection of structural classes.

### Construction of Fragment Class Hierarchy

Through applying the *Hierarchical Agglomeration* algorithm [51] to the BriX centroids, a fragment class hierarchy was constructed. The process can be described in three steps: (1) The coordinates of the centroids of fragment length 7 were collected; (2) A RMSD distance matrix was generated by employing the fast RMSD calculation method of Wolfgang Kabsch [49],[50]; (3) The final step consisted of a predefined number of iterations, which typified the desired levels in the hierarchy. In each iteration, the *Hierarchical Agglomeration* algorithm was called with an increasing distance threshold (Threshold  =  0.5 + k * 0.1, where *k* denotes de number of the iteration). By assembling the gathered results at each iteration (or level) a hierarchy of fragment classes was created.

### Protein Backbone Reconstruction Algorithm

#### Local fit approximation

Specifically, the algorithm can be described by an iteration process. At any time, the algorithm considers a determined position inside the protein structure, and attempts to identify a similar class centroid for that position. The selection procedure involves iteration over the possible fragment lengths. Large fragments that have a sufficiently small RMSD difference (resolution <1 Angstrom) compared to the original structure are favored. Whenever an appropriate centroid could not be found within the iteration, the fragment length was decreased by one residue. Otherwise, the respective BriX class was accepted as a solution and the procedure repeated for a following position in the protein structure. If a solution could not be obtained, the location in the structure was marked and the procedure repeated for the neighbouring position. The algorithm to reconstruct protein backbones using BriX classes can be described in pseudo code as follows:

For a given protein structure *X*

Create an empty solution set *S*

**FOR** position *i* = 1→*length*(*X*)

 Create an empty solution set for position *i s_i_*

 **FOR** fragment length *n* = 14→4

  **IF** *s_i_* = Ø

   Select fragment *Y* [*X_i_*−*X_i_*_+*n*_]

   **FOR** all fragment classes *Z* of length *n*

    **IF** *RMSD*(*fragment Y*, *centroid*(*Z*))<1*A*

     Add *Z* to *s_i_*

   *n* = *n*−1

  **ELSE**

   Continue

 Add *s_i_* to *S*

 *i* = *i*+1

**RETURN** S

#### Global fit approximation

As the local-fit approximation consists of looking for BriX classes that match local fragments of a target structure, the calculation of the global fit is less straightforward.

The number of possible sequences grows exponentially with the protein's target length. Therefore a strategy was necessary to prevent examining all sequences to output the best global-fit approximation. However, at any time, the algorithm should be able to backtrack to a previous solution when it gets stuck in a local minimum. Our algorithm follows a depth first search approach for memory-efficiency reasons. At any time, a solution queue keeps track of candidate structures created so far. These candidate structures, representing a partial approximation of the protein's backbone, are ordered in a way that the closest solutions are in front. In general terms, solutions with a longer candidate structure are favored over those with shorter reconstructions. To avoid a bias towards fragment length, the last fragment addition is not considered. When two candidate structures have the same size, the solution with the smallest distance between the last added fragment and the target structure is preferred. As long as the queue contains solutions and no solution has been found, the algorithm pops out the front candidate structure and tries to extend this structure using the best fragments matches in BriX. The selection of a good match is a two-step procedure: First, there is searched for local matches between the consecutive backbone segment, having an overlap of three residues with the already constructed candidate, and the BriX class centroids. Second, for every class match, a more specific search is carried out considering the fragments within this class after superimposing them on the target structure. For each class fragment that is in conformity with the preceding backbone reconstruction, i.e. when the RMSD between the overlapping residues is minimal (RMSD <0.3 Angstrom), a new extended candidate structure is put in the solution queue. When no viable fragment can be found, the algorithm tracks back to the second closest candidate in the solution queue.

We analyzed the efficiency of our algorithm on an Opteron (TM) Dual Core Processor 2.0 GHz. The execution time of this algorithm is inherently dependent on the size of the protein. For an average sized protein like the α G25K GTP-binding protein (see Figure 5C), for instance, the algorithm is able to output a solution within 5 minutes time.

All protein graphics in this article were generated with the YASARA software package [52] and PyMOL [53].

### Validation Datasets

For the creation of the BriX library, we used a list of 1,261 PDB chains downloaded from the WHAT IF website. These representative chains were collected from the Protein Data Bank on October 2002 using a sequence identity cutoff of 30%, a resolution higher than 2.1 Angstrom and an R factor less than 0.21.

For the plasticity results and the first validation test of BriX, 7,290 structures of the Astral set [42] were used. This set has a 1.8 Angstrom resolution and less than 40% internal structural homology. The structures were obtained directly from the Astral website.

The *human proteins* set was extracted from the PDB [54] by performing an advanced search. The 935 high-resolution structures from human origin were obtained by setting the *source organism* parameter to *Homo sapiens* and the *experimental method* parameter to *X-ray*.

### Plastic Sequences

As the sequence space within BriX was too small to perform the experiment with high reliability, the larger Astral40 set was used. For each fragment length (4–14 residues) groups containing fragments with identical sequences were created. Subsequently, the mutual RMSD of all fragment pairs within each group was calculated. To identify structural switches within the sequence, the distribution of the obtained RMSD values was plotted in a histogram with a bin size of 0.1 Angstrom.

## Supporting Information

Figure S1 Secondary structure composition of fragment classes. The DSSP secondary structure assignments for the four main secondary structure elements (helix, strand, turn and loop) were counted in fragment classes of 10 long (A) and 7 long (B) and plotted against the percentage of classes displaying this composition. Turns and loops mainly occur in 1 to 4 residue patterns, whereas helices and strands take up longer stretches within the fragment (peaks at 5 and 6 residues, respectively). Clearly shown is the presence of pure classes at length 7, i.e. classes that consist of only one secondary structure element. When a larger fragment length is considered the classes are generally composed of a mixture of elements.
(0.51 MB TIF)

Figure S2 Reconstruction of protein backbones using BriX classes. (A) Global fit of the Park & Levitt protein set. Shown is the distribution of RMSD observed after reconstructing the proteins present in the Park & Levitt set. The reconstruction algorithm was carried out in both directions: from N to C terminal (in red) and from C to N terminal (in blue). As the direction affected the outcome of the algorithm, the general RMSD distribution remained the same. With an average RMSD of 0.48 Angstrom to the crystallographic coordinates we improved previous obtained backbone reconstruction results. (B, C) Local fit of human protein backbones. These plots are the result of covering the human protein backbones and show data for all major SCOP classes: all α (in red), all β (in blue), a/β (in green) and a+β (in orange). The coverage experiment revealed that virtually the entire protein structure could be reconstructed by using BriX building blocks comprising 14 residues (B). At loop and turn locations smaller fragments (between 4 and 8 residues) are needed to describe their hypervariable nature (C).
(0.89 MB TIF)

Figure S3 Reconstruction bridging region between regular secondary structure elements and loop locations. The plots are the result of reconstructing the human protein backbones and show data for all major SCOP classes: all α (in red), all β (in blue), a/β (in green) and a+β (in orange). Shown are the local matches with BriX fragments for the regions between a regular secondary structure element and a loop region, where two residues belong to the regular side for the β Human C-reactive protein (A). The occurrences of local matches with regard to a particular length were counted and plotted in a histogram (B). As can be clearly seen, these regions are best approximated by smaller building blocks.
(2.00 MB TIF)
