# Supplementary figures and images for: Reconstruction of Protein Backbones from the BriX Collection of Canonical Protein Fragments
Source: PLoS Comput Biol. 2008 May 23;4(5):e1000083. doi: 10.1371/journal.pcbi.1000083 (PMC2367438; doi:10.1371/journal.pcbi.1000083)

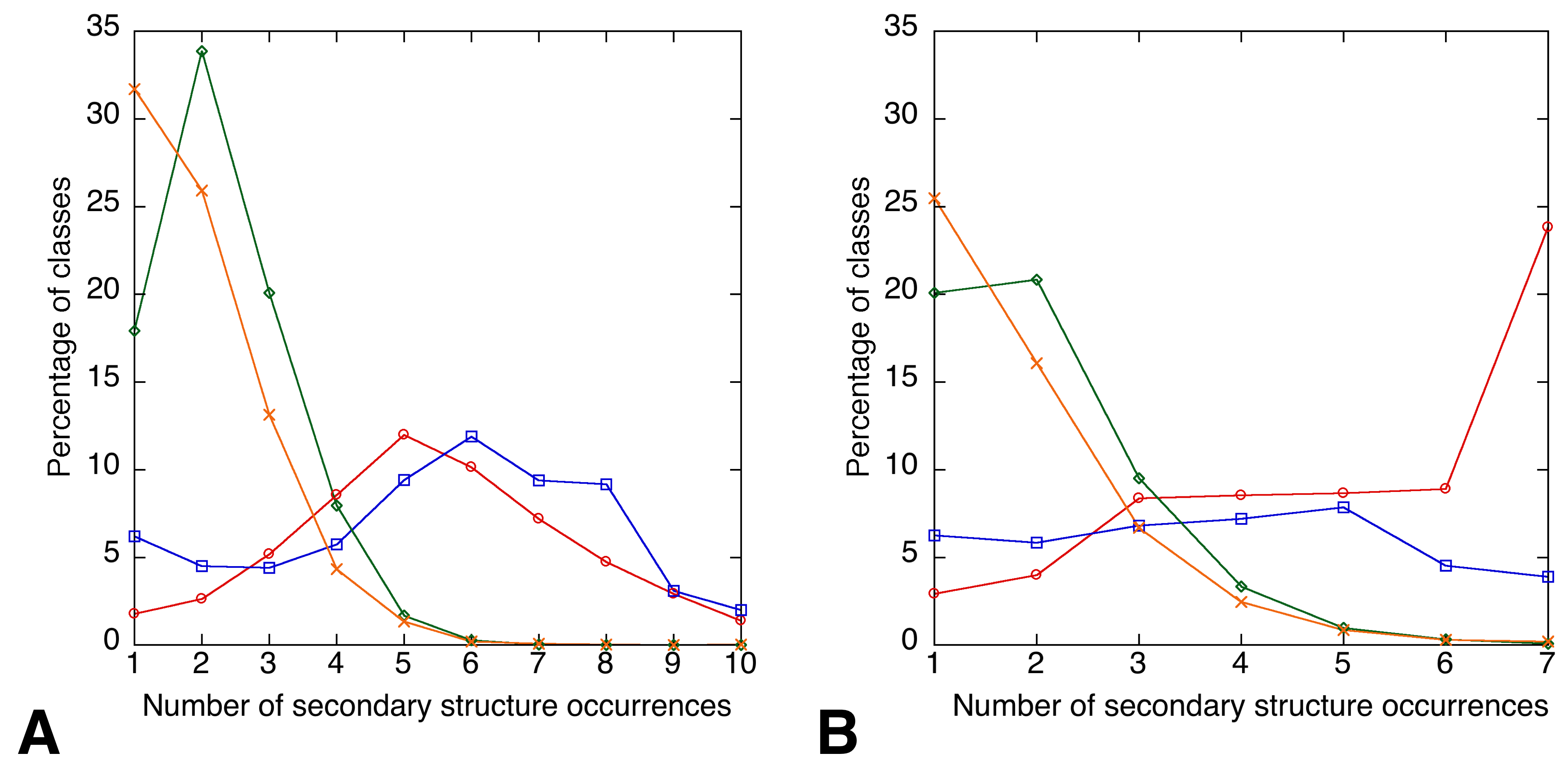

Supplement: Figure S1 — The DSSP secondary structure assignments for the four main secondary structure elements (helix, strand, turn and loop) were counted in fragment classes of 10 long (A) and 7 long (B) and plotted against the percentage of classes displaying this composition. Turns and loops mainly occur in 1 to 4 residue patterns, whereas helices and strands take up longer stretches within the fragment (peaks at 5 and 6 residues, respectively). Clearly shown is the presence of pure classes at length 7, i.e. classes that consist of only one secondary structure element. When a larger fragment length is considered the classes are generally composed of a mixture of elements. (0.51 MB TIF) [file pcbi.1000083.s001.tif]

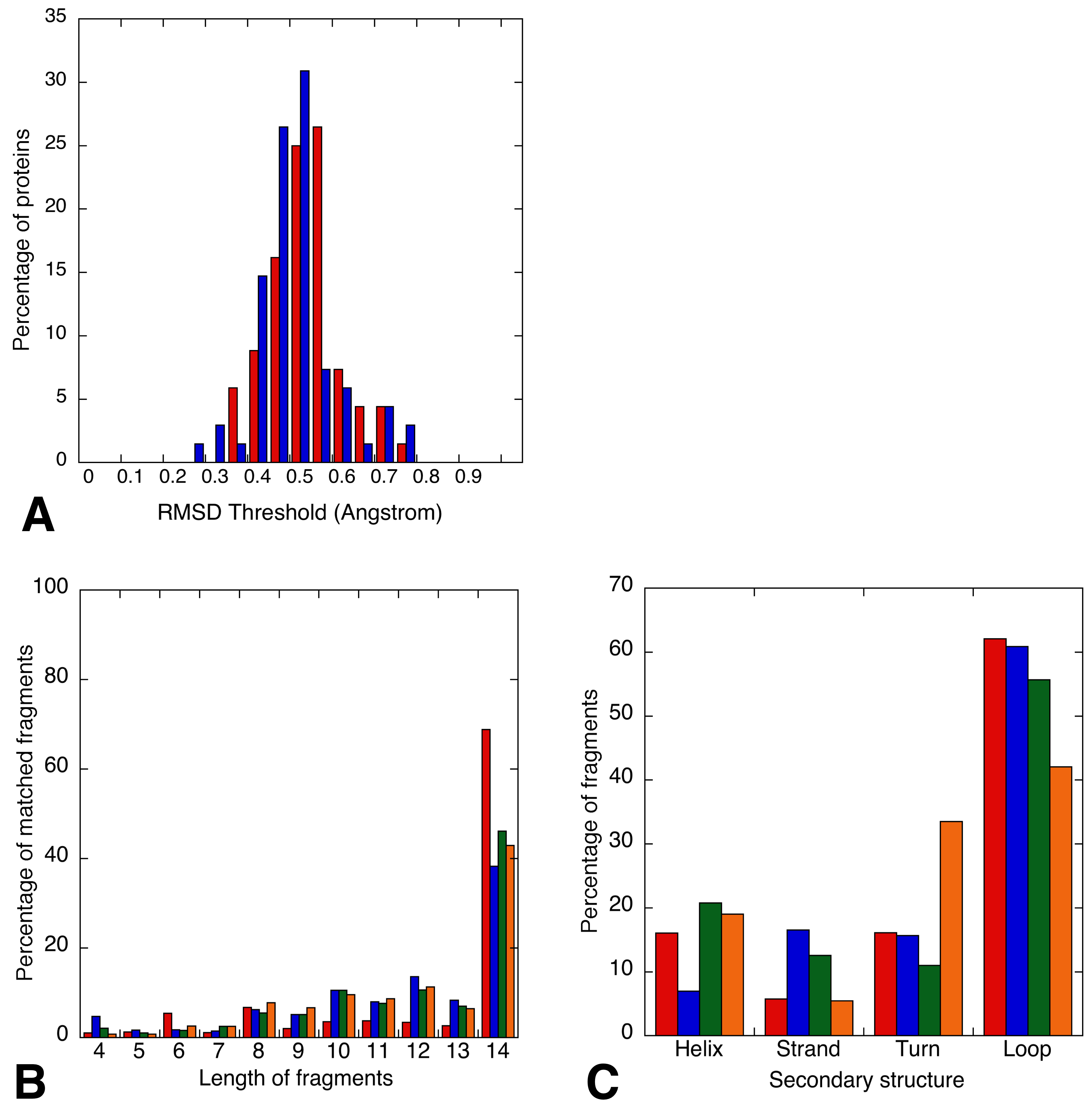

Supplement: Figure S2 — (A) Global fit of the Park & Levitt protein set. Shown is the distribution of RMSD observed after reconstructing the proteins present in the Park & Levitt set. The reconstruction algorithm was carried out in both directions: from N to C terminal (in red) and from C to N terminal (in blue). As the direction affected the outcome of the algorithm, the general RMSD distribution remained the same. With an average RMSD of 0.48 Angstrom to the crystallographic coordinates we improved previous obtained backbone reconstruction results. (B, C) Local fit of human protein backbones. These plots are the result of covering the human protein backbones and show data for all major SCOP classes: all α (in red), all β (in blue), a/β (in green) and a+β (in orange). The coverage experiment revealed that virtually the entire protein structure could be reconstructed by using BriX building blocks comprising 14 residues (B). At loop and turn locations smaller fragments (between 4 and 8 residues) are needed to describe their hypervariable nature (C). (0.89 MB TIF) [file pcbi.1000083.s002.tif]

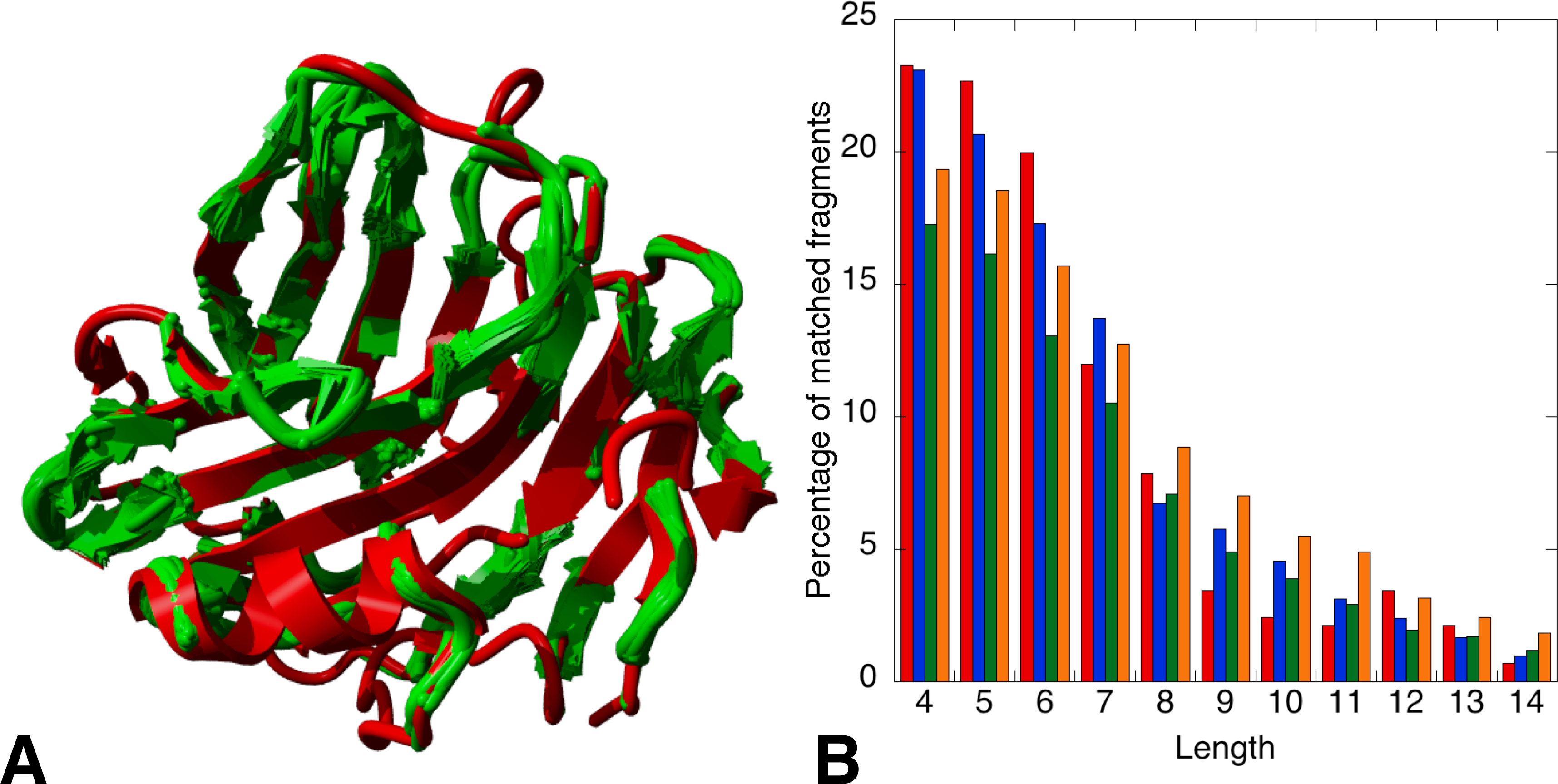

Supplement: Figure S3 — The plots are the result of reconstructing the human protein backbones and show data for all major SCOP classes: all α (in red), all β (in blue), a/β (in green) and a+β (in orange). Shown are the local matches with BriX fragments for the regions between a regular secondary structure element and a loop region, where two residues belong to the regular side for the β Human C-reactive protein (A). The occurrences of local matches with regard to a particular length were counted and plotted in a histogram (B). As can be clearly seen, these regions are best approximated by smaller building blocks. (2.00 MB TIF) [file pcbi.1000083.s003.tif]
